# Supplementary material for: MHC1-TIP enables single-tube multimodal immunopeptidome profiling and uncovers intratumoral heterogeneity in antigen presentation
Source: Commun Biol. 2026 Jan 22;9:296. doi: 10.1038/s42003-026-09570-6 (PMC12923572; doi:10.1038/s42003-026-09570-6)
Supplement: Supplementary file 2 — Supplemental Information [file 42003_2026_9570_MOESM2_ESM.pdf]

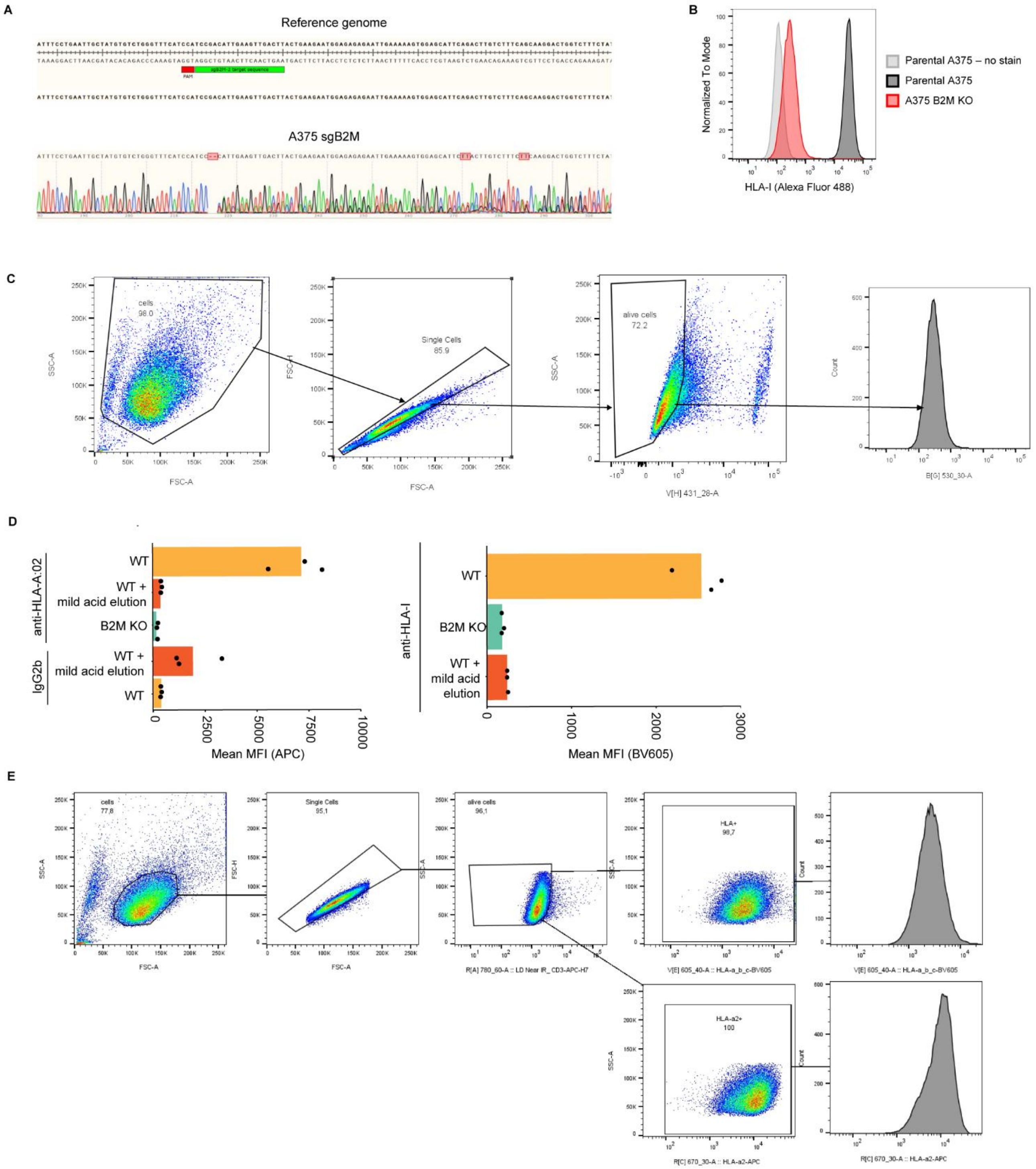

Supplementary Figure 1: Mild acid elution results in loss of cell surface MHC-I complexes

(A) Sanger sequencing Applied Biosystems Sequence Trace file alignment to sgB2M target sequence of the human reference genome hg38 in Snapgene v8.0.1.

(B) Flow cytometry staining with W6/32 antibody (pan-HLA-I).

(C) Gating strategy for (B).

(D) Flow cytometry staining with anti-pan-HLA-I (W6/32) and anti-HLA-A:02 including an isotype control using A375 wildtype cells, with and without mild acid treatment, and A375 B2M KO cells.

(E) Gating strategy for (D).

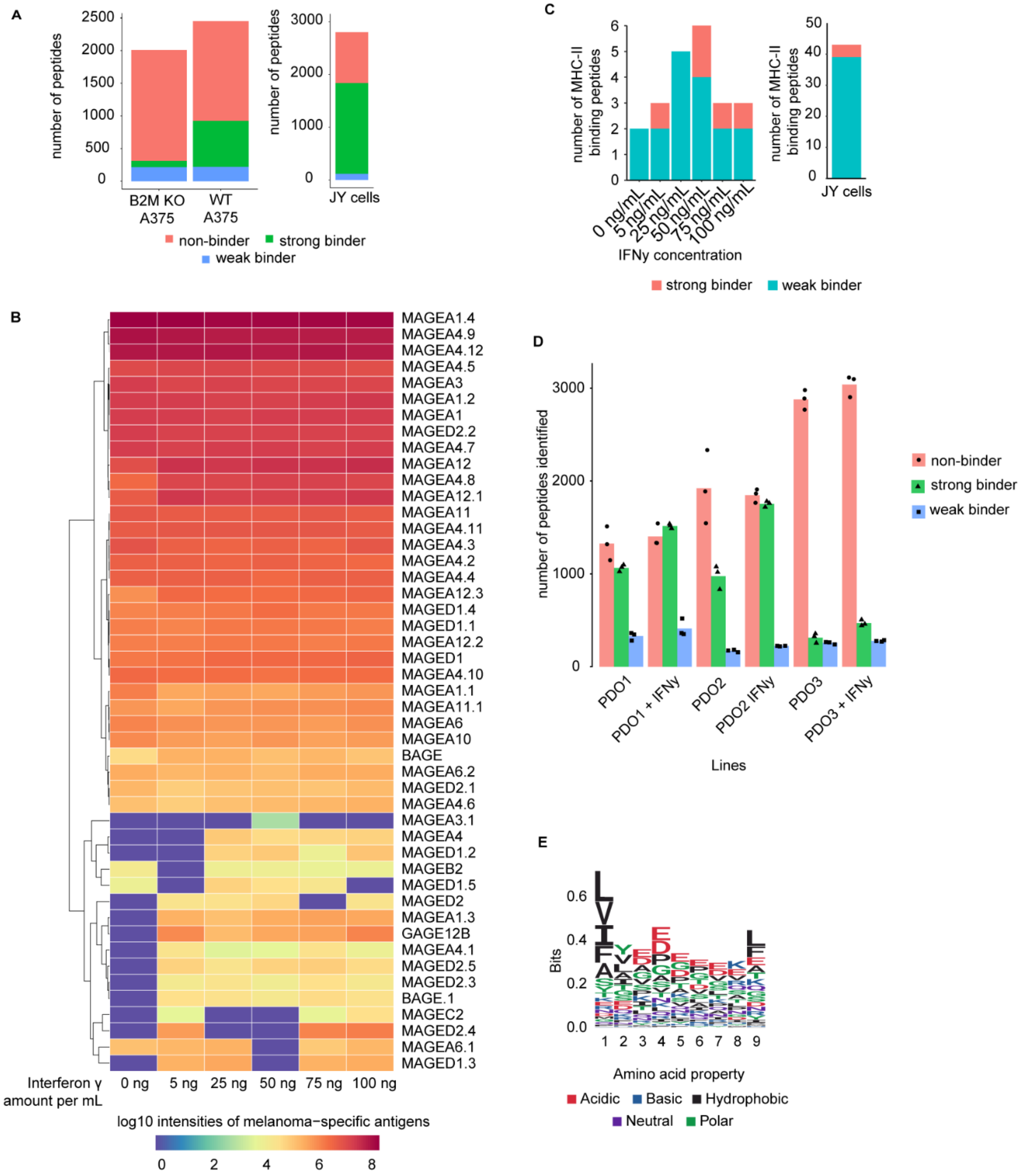

Supplementary Figure 2: MHC1-TIP shows specific recovery of MHC-I peptides in cell lines and patient-derived organoids

(A) Amount of predicted MHC class I binding peptides in 5 million A375 wildtype and B2M KO cells, and 10 million JY cells.

(B) Melanoma-specific antigens detected in A375 cells. Immunopeptides are labelled with the gene name of the parental protein of the antigen, conjugated with a numeric identifier for each peptide identified from that protein. Highlighted antigens were selected by matching their source protein name to MAGE, BAGE or GAGE.

(C) MHC class II predicted binders in A375 cells upon IFNγ treatment (1 million cells per concentration), and 10 million JY cells. The immunopeptidomes were measured in DDA mode.

(D) MHC class I predicted binders in patient-derived organoids, with and without IFNγ treatment (N=3 per organoid line).

(E) Sequence motif generated from peptides of length 9, eluted from PDO-1 after dispase treatment to dissociate the organoids.

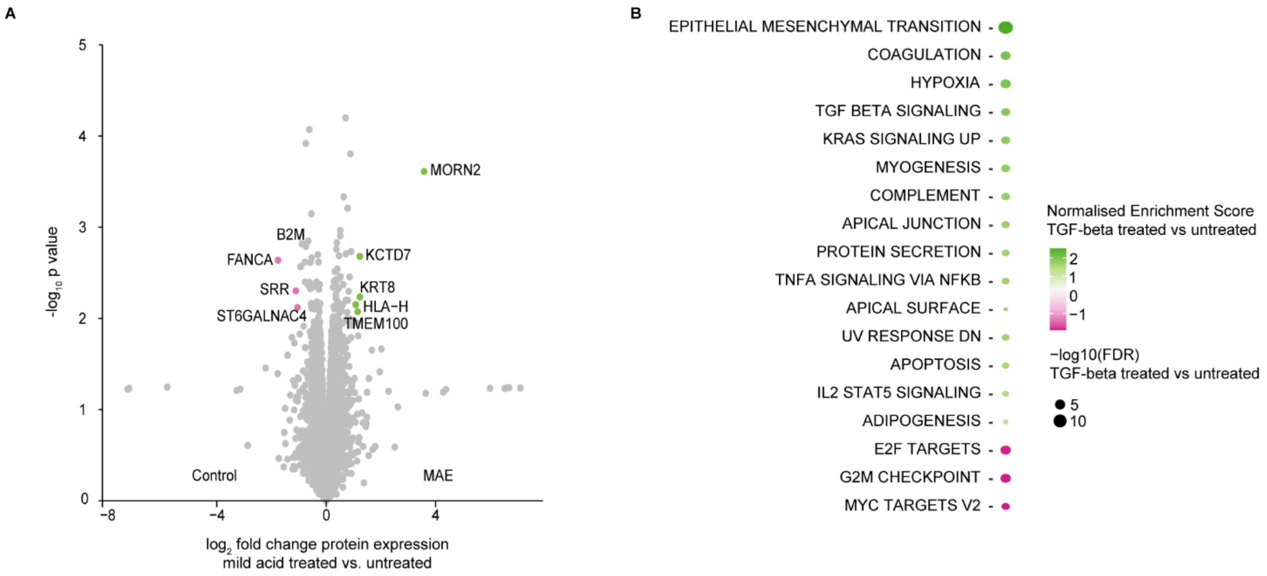

Supplementary Figure 3: MHC1-TIP enables multi-omic profiling

(A) Changes induced in the proteome after mild acid elution. Green dots represent significantly upregulated proteins (FDR < 0.05 and $\log_2$ fold change > 1) and pink dots represent significantly downregulated proteins (FDR < 0.05 and $\log_2$ fold change < -1)

(B) Significantly enriched pathways (FDR < 0.05) after gene set enrichment analysis using Hallmark gene sets

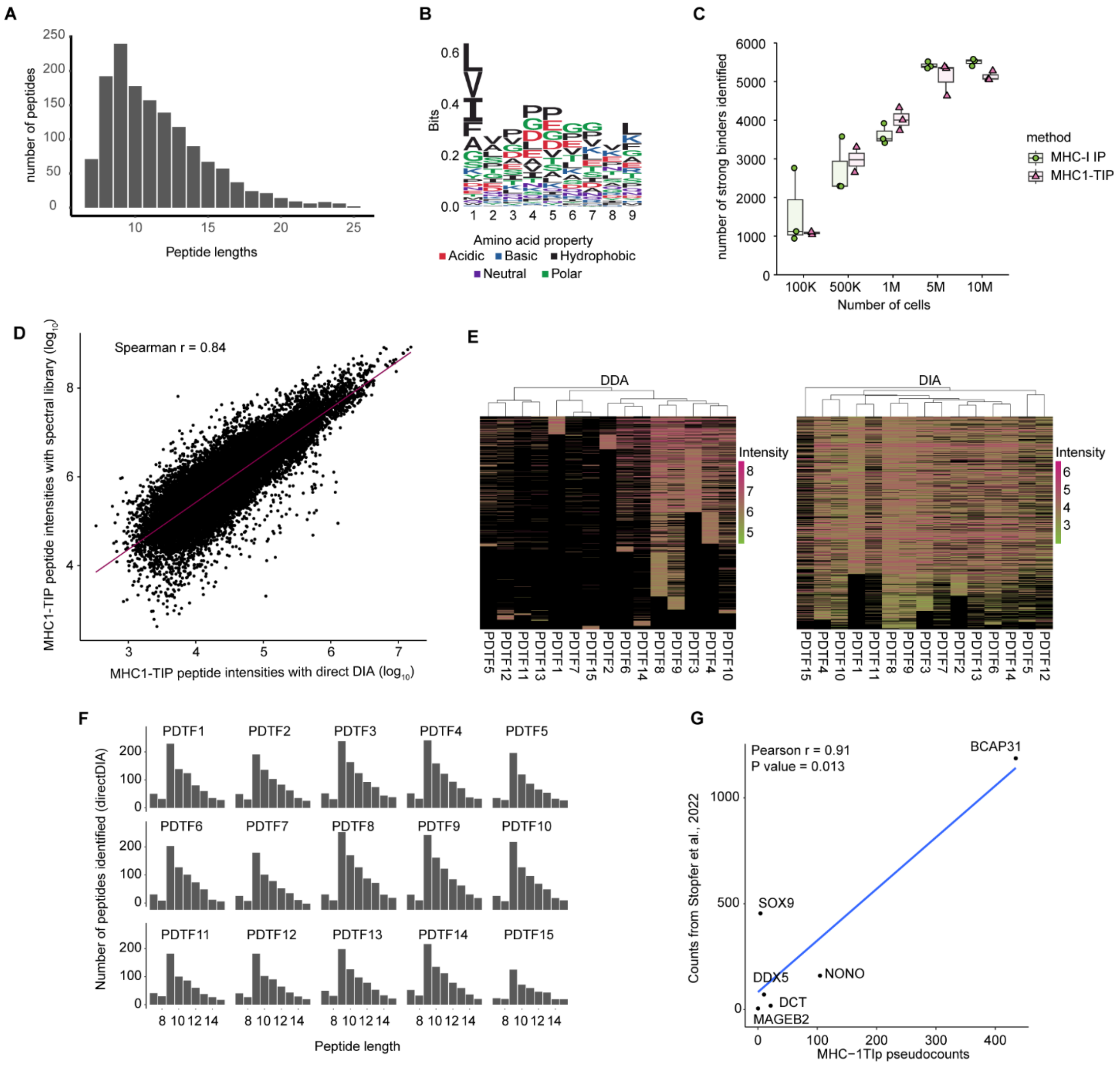

Supplementary Figure 4: MHC1-TIP enables quantitative immunopeptidomics from tumour tissue fragments using direct DIA

(A) Number of peptides detected across all 15 tissue fragments using DDA.

(B) Sequence motif of peptides of length 9 eluted from tissue fragments after dissociation of the fragment into a single-cell suspension using collagenase, DNAse and hyaluronidase.

(C) Direct DIA analysis of immunopeptidomes of A375 cells using MHC1-TIP and immunoprecipitation. The central lines in the shown boxplot are the median, the box shows the interquartile range, and the whiskers are extreme values upon removing outliers.

(D) Spearman correlation between peptide ion intensities quantified by direct DIA, and by DIA-NN with an experimentally generated spectral library, for all MHC1-TIP samples displayed in Figure 1E.

(E) Heatmaps showing data missing-ness (in black) with data-dependent and data-independent modes of acquisition from each tissue fragment. Intensities are plotted in log_{10} scale.

(F) Number of peptides detected per tissue fragments using direct DIA. All fragments show an

enrichment of peptides of length 9.

(G) Pseudocounts generated from MHC1-TIP data (immunopeptidomes of the untreated samples from the TGF-beta experiment; Figure 2) using a normalization method based on B2M protein copy numbers correlate (pearson correlation; two-sided t-test) with published hipMHC-quantified absolute antigen counts for the same cell type (A375) from Stopfer et al., 2022 (N=3 biologically independent samples).

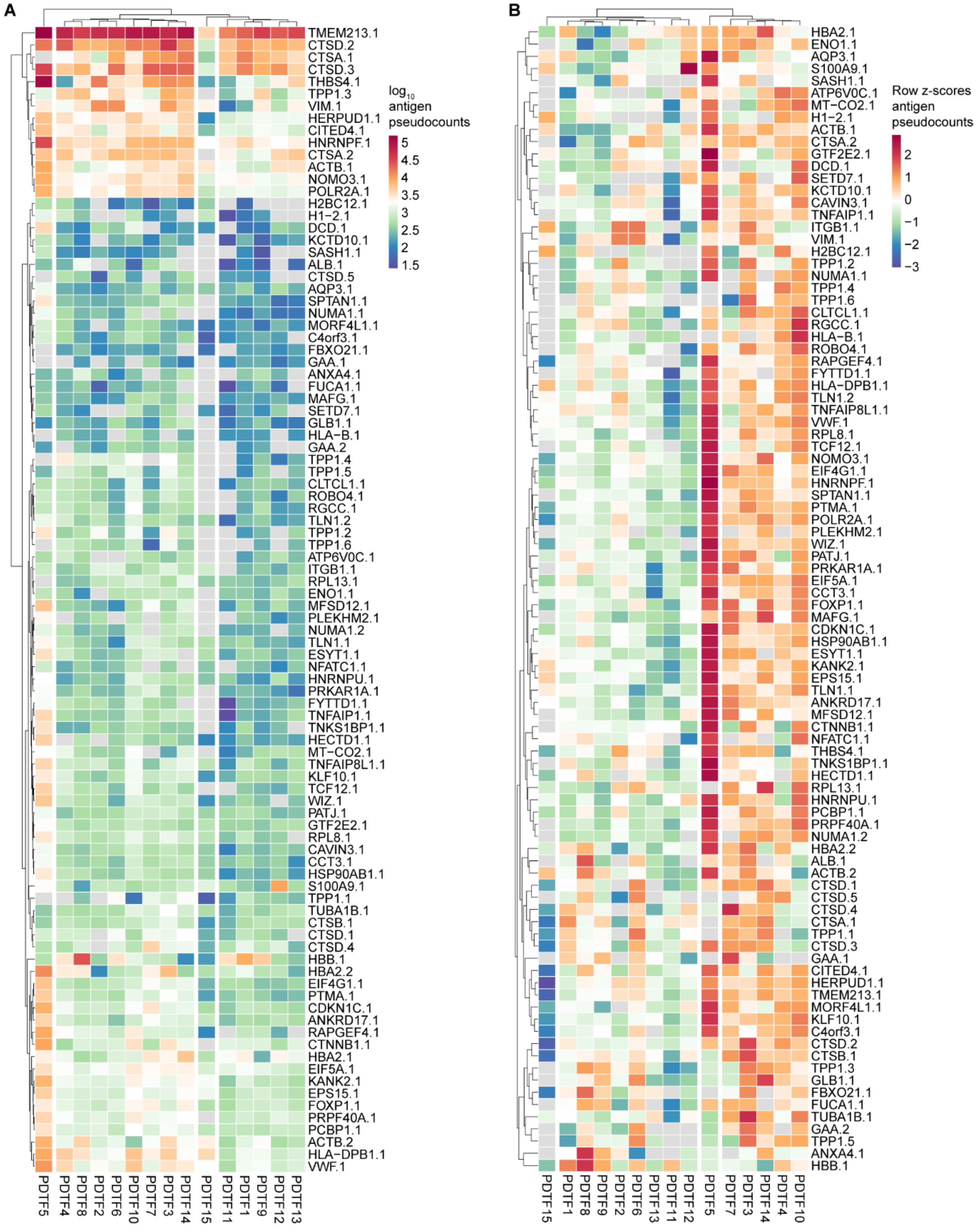

Supplementary Figure 5: Patient-derived tumour fragments show heterogeneity antigen abundances.

(A-B) Immunopeptides are labelled with the gene name of the parental protein of the antigen, conjugated with a numeric identifier for each peptide identified from that protein.

(A) Heatmap displaying pseudocounts of antigen per cell in each tissue fragment. Detected antigen copy numbers per cell range 4 orders of magnitude.
(B) Scaling and centering antigen pseudocounts row-wise reveals heterogeneity in antigen presentation across the fragments.
