# Supplementary material for: MHC1-TIP enables single-tube multimodal immunopeptidome profiling and uncovers intratumoral heterogeneity in antigen presentation
Source: Commun Biol. 2026 Jan 22;9:296. doi: 10.1038/s42003-026-09570-6 (PMC12923572; doi:10.1038/s42003-026-09570-6)
Supplement: Supplementary file 3 — Reporting summary [file 42003_2026_9570_MOESM3_ESM.pdf]

nature portfolio

Corresponding author(s): Rik G.H. Lindeboom

Last updated by author(s): Dec 19, 2025

Reporting Summary

Nature Portfolio wishes to improve the reproducibility of the work that we publish. This form provides structure for consistency and transparency in reporting. For further information on Nature Portfolio policies, see our Editorial Policies and the Editorial Policy Checklist.

Statistics

For all statistical analyses, confirm that the following items are present in the figure legend, table legend, main text, or Methods section.

n/a	Confirmed	
☐	☒	The exact sample size (*n*) for each experimental group/condition, given as a discrete number and unit of measurement
☐	☒	A statement on whether measurements were taken from distinct samples or whether the same sample was measured repeatedly
☐	☒	The statistical test(s) used AND whether they are one- or two-sided<br>*Only common tests should be described solely by name; describe more complex techniques in the Methods section.*
☒	☐	A description of all covariates tested
☒	☐	A description of any assumptions or corrections, such as tests of normality and adjustment for multiple comparisons
☐	☒	A full description of the statistical parameters including central tendency (e.g. means) or other basic estimates (e.g. regression coefficient) AND variation (e.g. standard deviation) or associated estimates of uncertainty (e.g. confidence intervals)
☐	☒	For null hypothesis testing, the test statistic (e.g. *F*, *t*, *r*) with confidence intervals, effect sizes, degrees of freedom and *P* value noted<br>*Give P values as exact values whenever suitable.*
☒	☐	For Bayesian analysis, information on the choice of priors and Markov chain Monte Carlo settings
☒	☐	For hierarchical and complex designs, identification of the appropriate level for tests and full reporting of outcomes
☐	☒	Estimates of effect sizes (e.g. Cohen's *d*, Pearson's *r*), indicating how they were calculated

Our web collection on statistics for biologists contains articles on many of the points above.

Software and code

Policy information about availability of computer code

Data collection	Mass spectrometry data was acquired using Xcalibur version 4.2.
Data analysis	Mass spectrometry data was analysed using FragPipe versions 21, 22 and 23, or using DiaNN version 2.0. All custom code for downstream analysis is available on the GitHub repository associated to this manuscript: https://github.com/LindeboomLab/MHC1-TIP.<br>Flow cytometry data was analyzed using FlowJo 10.10.0.

For manuscripts utilizing custom algorithms or software that are central to the research but not yet described in published literature, software must be made available to editors and reviewers. We strongly encourage code deposition in a community repository (e.g. GitHub). See the Nature Portfolio guidelines for submitting code & software for further information.

Data

Policy information about availability of data

All manuscripts must include a data availability statement. This statement should provide the following information, where applicable:

- Accession codes, unique identifiers, or web links for publicly available datasets
- A description of any restrictions on data availability
- For clinical datasets or third party data, please ensure that the statement adheres to our policy

The data presented in this study is available through the PRIDE partner repository under accession number PXD065279.

Research involving human participants, their data, or biological material

Policy information about studies with human participants or human data. See also policy information about sex, gender (identity/presentation), and sexual orientation and race, ethnicity and racism.

Reporting on sex and gender	NA
Reporting on race, ethnicity, or other socially relevant groupings	NA
Population characteristics	NA
Recruitment	NA
Ethics oversight	NA

Note that full information on the approval of the study protocol must also be provided in the manuscript.

Field-specific reporting

Please select the one below that is the best fit for your research. If you are not sure, read the appropriate sections before making your selection.

☒ Life sciences ☐ Behavioural & social sciences ☐ Ecological, evolutionary & environmental sciences

For a reference copy of the document with all sections, see nature.com/documents/nr-reporting-summary-flat.pdf

Life sciences study design

All studies must disclose on these points even when the disclosure is negative.

Sample size	No sample size calculation was performed. Matched proteomes and immunopeptidomes from cell line or organoid cultures where statistical testing was performed were measured in technical triplicates, as this is considered the standard in controlled in vitro experiments, where at least 3 measurements are needed to estimate variability and perform statistical analysis. For measurements on tumor fragments, we profiled all obtained fragment from the tumor sample, and sample size was thus determined by the size of the tumor.
Data exclusions	Due to a technical issue one matched proteome from untreated A375 cells in the TGFb pertubation experiment needed to be excluded.
Replication	All measurements where a statistical test was performed at least in triplicate, with the exception of one matched immunopeptidome/ proteome sample after TGFb treatment in A375 cells. Due to a technical issue, one of the samples needed to be excluded. Immunopeptidomes generated by our new MHC1-TIP workflow are shown to highly reproducible in our manuscript.
Randomization	No randomization was performed in this study. All comparisons were performed on in vitro samples where no covariates were present.
Blinding	Blinding was not applicable because the experimental conditions in our cell culture experiments were controlled by the researcher, and there is no subjective interpretation during data collection using mass spectrometry.

Reporting for specific materials, systems and methods

We require information from authors about some types of materials, experimental systems and methods used in many studies. Here, indicate whether each material, system or method listed is relevant to your study. If you are not sure if a list item applies to your research, read the appropriate section before selecting a response.

Materials & experimental systems

n/a	Involved in the study	
☐	☒	Antibodies
☐	☒	Eukaryotic cell lines
☒	☐	Palaeontology and archaeology
☒	☐	Animals and other organisms
☒	☐	Clinical data
☒	☐	Dual use research of concern
☒	☐	Plants

Methods

n/a	Involved in the study	
☒	☐	ChIP-seq
☒	☐	Flow cytometry
☒	☐	MRI-based neuroimaging

Antibodies

Antibodies used	For IP-based immunopeptidomics, we made use of in-house generated clone W6/32 anti-HLA-A/B/C antibody, purified from a hybridoma cell line originally obtained as ATCC HB-95. W6/32 conjugated to BV605 was obtained from BioLegend (cat no. 311432), anti-HLA-A2 conjugated to APC from clone BB7.2 from BD Biosciences (cat. no. 561341) and IgG2b conjugated to APC from BioLegend (cat. no. 402205).
Validation	In addition to extensive validation in literature, this manuscript contains validation data for W6/32 anti-HLA-A/B/C antibody through B2M knock out.<br>Validation data for anti-HLA-A2-APC, and W6/32-BV605 is provided by the suppliers at https://www.bdbiosciences.com/en-eu/products/reagents/flow-cytometry-reagents/research-reagents/single-color-antibodies-ruo/apc-mouse-anti-human-hla-a2.561341?tab=product_details, and https://www.biolegend.com/en-ie/products/brilliant-violet-605-anti-human-hla-a-b-c-antibody-12842?GroupID=BLG5954, respectively.

Eukaryotic cell lines

Policy information about cell lines and Sex and Gender in Research

Cell line source(s)	A375 cells were given by Prof. Reuven Agami, and JY cells by Prof. Ton Schumacher. Both cell lines were originally obtained from ATCC.
Authentication	None of the cells used were authenticated.
Mycoplasma contamination	All of the cells used tested negative for mycoplasma contamination in PCR-based mycoplasma tests performed once in two months.
Commonly misidentified lines<br>(See ICLAC register)	No commonly misidentified cell lines were used in this study.

Plants

Seed stocks	*Report on the source of all seed stocks or other plant material used. If applicable, state the seed stock centre and catalogue number. If plant specimens were collected from the field, describe the collection location, date and sampling procedures.*
Novel plant genotypes	*Describe the methods by which all novel plant genotypes were produced. This includes those generated by transgenic approaches, gene editing, chemical/radiation-based mutagenesis and hybridization. For transgenic lines, describe the transformation method, the number of independent lines analyzed and the generation upon which experiments were performed. For gene-edited lines, describe the editor used, the endogenous sequence targeted for editing, the targeting guide RNA sequence (if applicable) and how the editor was applied.*
Authentication	*Describe any authentication procedures for each seed stock used or novel genotype generated. Describe any experiments used to assess the effect of a mutation and, where applicable, how potential secondary effects (e.g. second site T-DNA insertions, mosiacism, off-target gene editing) were examined.*
